# The Disturbing Effect of Neuromuscular Fatigue on Postural Control Is Accentuated in the Premenstrual Phase in Female Athletes

**DOI:** 10.3389/fphys.2021.736211

**Published:** 2021-10-18

**Authors:** Maissa Kacem, Rihab Borji, Sonia Sahli, Haithem Rebai

**Affiliations:** Research Laboratory: Education, Motricité, Sport et Santé, EM2S, LR19JS01, High Institute of Sport and Physical Education of Sfax, University of Sfax, Sfax, Tunisia

**Keywords:** athletes, static postural control, dynamic postural control, menstrual cycle, muscle fatigue

## Abstract

This study explored the fatigue effect on postural control (PC) across menstrual cycle phases (MCPs) in female athletes. Isometric maximal voluntary contraction (IMVC), the center of pressure sway area (CoParea), CoP length in the medio-lateral (CoP_LX_) and antero-posterior (CoP_LY_) directions, and Y-balance test (YBT) were assessed before and after a fatiguing exercise during the follicular phase (FP), mid-luteal phase (LP), and premenstrual phase (PMP). Baseline normalized reach distances (NRDs) for the YBT were lower (*p* = 0.00) in the PMP compared to others MCPs, but the IMVC, CoParea, CoP_LX_, and CoP_LY_ remained unchanged. After exercise, the IMVC and the NRD decrease was higher at PMP compared to FP (*p* = 0.00) and LP (*p* = 0.00). The CoParea, CoP_LX_, and CoP_LY_ increase was higher in the PMP compared to FP (*p* = 0.00) and LP (*p* = 0.00). It was concluded that there is an accentuated PC impairment after exercise observed at PMP.

## Introduction

Postural control (PC) is fundamental for human motor abilities not only to perform daily living activities (Taheri et al., 2019; Sabashi et al., 2021), but also to perform a high level of physical performance during sports training or competition (Geddam et al., 2014). PC assessment was usually conducted in athletes to identify or to prevent the risk of musculoskeletal injuries (Yim et al., 2018). In this context, it has been documented that postural balance impairments may affect physical performance (Zemková, 2014) and increase the risks for sports injuries (Paterno et al., 2010). Importantly, it has been reported that female athletes are more prone to the risk of injuries, such as anterior cruciate ligament injuries and lateral ankle sprains, compared to males while practicing the same sports activities (Ristolainen et al., 2009; Stijak et al., 2015). This higher injury rate in females compared to males has been linked to hormonal differences and fluctuations (Hewett et al., 2007). To this end, previous studies explored PC in women through different menstrual cycle phases (MCPs) governed by hormonal changes, but results remain controversial (Ekenros et al., 2011; Lee and Yim, 2016; Lee et al., 2017). In fact, in sedentary women, greater postural sway during or around ovulation compared to the other phases were observed (Petrofsky and Lee, 2015; Lee and Yim, 2016; Lee et al., 2017). As well, impaired static PC in the luteal phase (LP) was found in moderately active women with premenstrual symptoms (PMS), but not in those without PMS (Fridén et al., 2003, 2005; Ekenros et al., 2011). Kaya and Çelenay (2016) indicated that the worst dynamic PC was during the menses [early follicular phase (FP)], when estrogen level was low, compared to mid-FP and mid-LP in active women. However, other studies did not find any significant effect of MCPs on PC (Fridén et al., 2005; Hertel et al., 2006; Abt et al., 2007; Ekenros et al., 2011; Ericksen and Gribble, 2012).

PC results from the integration of sensory information (visual, vestibular, and somatosensory) by the central nervous system (CNS) to recruit adequately the muscles (Horak, 2009). Hence, it could be impaired by the dysfunction in any part of these systems (Horak, 2009). Accordingly, it has been shown that neuromuscular fatigue of the lower limb muscles impairs PC (Konstantopoulos et al., 2021). It seems that neuromuscular fatigue impairs the muscle mechanical properties (Bilodeau et al., 2001) and the proprioceptive system (Hiemstra et al., 2001) required for postural balance regulation. In addition, it has been reported that PC is deteriorated due to both local and general fatigue, increasing the risk for traumatic musculoskeletal injuries (Gribble and Hertel, 2004; Pau et al., 2014). Since athletic performances need simultaneously musculoskeletal system integrity and well-developed PC (Ohlendorf et al., 2020), investigating the effect of neuromuscular fatigue on PC in athletes has been an interest for many studies (Thiele et al., 2015; Troester and Duffield, 2019). In this context, PC was shown to be more disturbed by neuromuscular fatigue in females compared to males (Gribble and Hertel, 2004; Whyte et al., 2015). Muscle force, as well as neuromuscular fatigue, could be also influenced by MCP. In fact, it has been found that the muscle force and endurance were higher during the FP compared to LP and menstrual phase (Pallavi et al., 2017). Recently, higher peripheral fatigue, associated with higher muscle damage, was observed in the premenstrual phase (PMP) compared to the MCP (Graja et al., 2020).

While the deteriorating effects of neuromuscular fatigue and MCP on PC have been widely explored independently, to our best knowledge, no previous study has been focused on the interaction effects of these factors on PC in female athletes. In fact, one of the most sport performance limits in female athletes is the higher knee injury rate specifically involving the anterior cruciate ligament compared to male athletes (Lee et al., 2013; Stijak et al., 2015), and the main underlying factor of this higher injury rate is the altered PC (Kjær and Hansen, 2008) linked to the hormonal fluctuations (Hewett et al., 2007). Both MC-related postural impairments and neuromuscular fatigue were known to result in increased sport-related injury risk. Thus, there is a great need for extending research in this area as the alterations in neuromuscular control may result in poorer control of movement, leading to an increased risk of non-contact lower limb injuries. Athletes and coaches would better be counseled regarding the MC effects on neuromuscular fatigue, PC, and risk of injury. By investigating the effect of MC hormonal fluctuations on PC following a fatiguing exercise, female athletes, coaches, and medical professionals could better understand the risk factors and find successful interventions for limiting this eventual impairment. To this end, they can suggest periodization of training programs for female athletes to take advantage of any optimal hormonal fluctuations and reduce the risk of musculoskeletal injury. Therefore, our study aimed to investigate the effect of MCP on static and dynamic PC after high-intensity fatiguing exercise in eumenorrheic female handball players.

## Materials and Methods

### Participants

The sample size was *a priori* calculated as suggested by Beck (2013) using the software G^*^power for Windows (version 3.1.9.2; Heinrich Heine University Düsseldorf, Northrhine-Westphalia, Germany) as recommended by Faul et al. (2007) to determine the number of participants necessary to identify PC changes with fatigue. For computing sample size, we used the effect size Cohen's *f* (calculated based on a partial $\eta_{\text{p}}^{2}$) estimated at 0.86 based on a previously published work by Boyas et al. (2013). Values for an alpha, power, correlation among repeated measures, and the non-sphericity correction (ε) were set at 0.05, 0.8, 0.5, and 1, respectively. In total, to reach the desired power, data from at least four participants were deemed to be sufficient to minimize the risk of Type II statistical error. To accommodate a possible dropout of some participants, we recruited 12 female athletes. Our recruitment strategy consisted of a three-stage screening process to delineate the sample. In the first stage, we randomly selected 22 female handball players from the regional senior handball team. Seven out of these 22 players were excluded from the study because they did not meet all inclusion criteria. Females included were handball players from the regional senior handball team and had a regular menstrual cycle (28 ± 1 day). Exclusion criteria were clinical ankle instability, vestibular or visual impairments, lower limb musculoskeletal injury in the previous 6 months, premenstrual syndromes, pregnancy, contraception or hormonal supplementary, and injections through the previous 3 months. In the second stage, 15 out of a total number of players who met the inclusion criteria were selected. Three of the screened players did not accomplish the three test sessions for different causes. As a result, 12 female team handball players (age: 21.0 ± 1.6 years, height: 1.72 ± 0.05 cm, weight: 65.0 ± 5.6 kg, weekly training volume: 11 ± 1 h, training experience: 6 ± 1 years) with the regular menstrual cycle (28 ± 1 day) participated in this study. They practiced in five training sessions of 2 h each and participated in one competitive match weekly in the regional senior handball series. Following an explanation of all the experimental procedures as well as their risks and benefits, all participants provided their written informed consent prior to the experiment. The study protocol was conducted in accordance with the Declaration of Helsinki and approved by the Southern Committee Protection of Persons (CPP SUD: 0042/2017).

### Experimental Design

Participants were invited to come four times to the institute laboratory. The first session was opted to familiarize participants with the experimental protocol including isometric maximal voluntary contraction (IMVC), static and dynamic balance tests, and the fatiguing exercise. This session was performed 3 days before the experimental protocol execution to eliminate any fear of using new equipment, and to ensure high-quality results. Moreover, anthropometric measurements were performed in this familiarization session.

The three test sessions corresponded to the three MCPs: FP (day 13), LP (day 21), and PMP (day 27). These three sessions were conducted in a randomized crossover design. In fact, some athletes started the first session in the FP. However, others started in the LP or PMP during the same experimental month. MCP was verified by an ovulation test on day 12–13 of the menstrual cycle. At each session, participants were required to successively perform postural tests, 5 min warm-up, three IMVC, the fatiguing exercise, an IMVC, and the postfatigue postural tests (Bizid et al., 2009; Chaubet et al., 2012; da Costa et al., 2019; Martins et al., 2020). For each test, the mean of the three preexercise trials was calculated and then statistically analyzed as the baseline value. It is necessary to achieve all tests in a short time period after the fatiguing exercise to avoid recovery, and based on previous studies investigating the effect of fatigue on muscle force and PC, only one trial for each test was performed at the postfatigue condition (Boyas et al., 2011; Bisson et al., 2014; Larson and Brown, 2018). The center of pressure (CoP) measurement and the Y-balance test (YBT) trials were randomized both in the pre and postexercise conditions. For all measurements, all participants were able to perform correctly the tasks in both prefatigue and postfatigue conditions. We did not have any discard/ repeat failed trials. The whole postfatigue test procedure (Zech et al., 2012) did not exceed 4 min as it has been previously demonstrated that PC is still significantly affected until 8 min postfatigue (Lin et al., 2009). The assessors were specialists who had significant experience in the physical activity field. All PC tests were taken by the same examiner, a PhD expert in balance measurement, and all MVCs measurements were made by the same PhD expert in dynamometric assessment.

### MCP Discrimination

Rectal temperature was recorded every morning before arising from bed for three consecutive months before and during the experiment. The beginning of the FP was indicated by the onset of menses, and the beginning of ovulation was indicated by an increase in temperature of 0.5°C. In addition, on days 12 and 13 of the menstrual cycle, we used the ovulation test strips (OVU Test Pro LH) measuring the luteinizing hormone (LH) surge meaning that the ovulation was reached within 24–48 h after detection of the LH surge, then the LP started.

### Muscle Force Assessments

Participants practiced a 5-min warm-up consisting of several submaximal contractions of knee extension muscles at a self-selected intensity. Then, they performed three IMVCs of the dominant leg (the leg used to kick the ball) knee extensor muscles under strong verbal encouragement. They were seated on an isometric dynamometer (Good Strength, Metitur, Finland) equipped with a cuff attached to a strain gauge. The hip and knee angles were set at 90° during all measurements. The highest value of these three IMVCs was considered as the reference IMVC to calculate the 80% IMVC for the fatiguing exercise. IMVC was tested once again immediately after the fatiguing exercise.

### PC Assessment

#### Posturographic Measurements

Posturography is a method that assesses postural stability (Toppila et al., 2006), which is considered to be the gold standard for postural balance assessment (Huurnink et al., 2013). Moreover, it is non-invasive, objective, rapid, and easy to administer. The CoP excursions were collected before and after the fatiguing exercise using a static stabilometric platform (PIMVCostureWin, Techno Concept, Cereste, France; 12-bit A/D conversion), composed of a steel plate supported by three strain gauges. The CoP excursions were collected at a sampling rate of 40 Hz with respect to the French Association of Posturography recommendations (Association Française de Posturologie, 1985). The platform was leveled with the surrounding floor and placed at a 3 m distance from the dynamometer. Participants were asked to stand as still as possible on the force platform with barefoot on one leg (dominant leg) while the other leg flexed by 45° at the hip and knee so as to resemble the starting position of a front kick. Their arms are comfortably placed downward at either side of the body. A plastic device was provided with the platform to maintain the same foot positions for all the measurements. This posturographic test was performed in two visual conditions. In the eyes open (EO) condition, participants were instructed to look straight ahead at a white cross placed onto the wall 2 meters away at eye level. In the EC conditions, in order to ensure the absence of visual information and to perform the same task, they wore a blindfold. They were asked to keep their gaze horizontal in a straight-ahead direction. Trials were randomized to eliminate learning or fatigue effects. Three trials were performed in each experimental condition for each participant before the fatiguing exercise whereas only one trial was performed for the postfatigue assessment. Each trial lasted 25.6 s (French Posturography Association norms) and 1 min of rest was allowed between two consecutive trials. Data collection was initiated after participants adopted the required posture on the platform, stabilized their postural sway, and signaled the experimenter that they were ready to begin. Then, the CoP excursions were computed from the ground reaction forces and their associated torques. During the upright standing postures, participants oscillate with their body relatively rigid, and the reaction force applied to the body is relatively constant. Thus, the associated torque variations depend mainly on the CoP excursions (Asseman et al., 2008). The muscular torque that controls the body oscillations is represented by CoP excursions (Winter et al., 1998; Asseman et al., 2008). CoP signals were smoothed using a second-order Butterworth filter with a 10 Hz low-pass cutoff frequency. To evaluate the static PC of our participants, three CoP sways parameters were analyzed in this study: The CoParea (CoParea; 90% confidence ellipse) was analyzed to evaluate the stability performance of participants. The lower the CoP sway, the better the PC will be (Caron et al., 2000). Furthermore, the CoP lengths corresponding to the sum of CoP displacement in the medio-lateral (CoP_LX_) and in antero-posterior (CoP_LY_) directions reflecting the topographical features of the plantar pressure distribution (Han et al., 1999) were analyzed. These parameters were averaged for the three trials per condition and per participant for statistical analysis. In case of a failed trial, data collection was stopped and the trial was repeated. A trial was considered as a failure if the participant put the contralateral foot down, stepped out of position, changed her feet or arms from the starting position, touched something for support, missed the stopwatch pad, or lost balance before completing the 25.6 s (Alsubaie et al., 2019).

### Dynamic PC Assessment

The YBT is a commercially available tool for assessing dynamic PC, and it possesses excellent intra-tester (0.85–0.89) and inter-tester (0.97–1.00) reliability (Plisky et al., 2009). In each MCP, athletes performed the YBT with three trials before exercise to consider the mean as the preexercise value and only one trial immediately after exercise. This test was performed by the dominant leg randomly in three directions derived from the Star Excursion Balance Test (SEBT): the anterior, the posteromedial, and the posterolateral directions. This test is considered to be an indicator of lower limb injury risk (Plisky et al., 2006; Smith et al., 2015). Participants have to maintain their balance on the dominant leg while sliding a block as far as possible in each direction with the contralateral leg. The criteria denoting a failed trial were chosen in line with previously published literature. A trial was discarded and repeated if participants (i) took the weight on the reaching foot; (ii) failed to bring back the reaching foot to the starting position without losing control; (iii) failed to keep both hands on hips; (iv) failed to keep the stance foot at the same place; or (v) kicked the reach-block to gain more distance (Plisky et al., 2009). Measures of the YBT reach distances were normalized for leg length to calculate the normalized reach distance (NRD) using the following formula:


$$\begin{array}{l} NRD\;\left(\%\right)=\left[Reach\;Distance\;\left(cm\right)/\;Leg\;Length\;\left(cm\right)\right]\;\times \;100 \end{array}$$

Leg length was obtained by measuring the distance between the anterior-superior iliac spine and the most distal aspect of the medial malleolus (Gribble and Hertel, 2003). A composite score of the overall YBT was calculated by averaging the individual normalized scores for each direction (Butler et al., 2013).

### Fatiguing Exercise

Our fatigue protocol is a modified version from that previously described by Smith-Ryan et al. (2014). Participants were required to follow their force production on a computer screen placed in front of them, which displayed their real-time digitized force signal. Visual feedback was provided on the screen at 80% of their IMVC with a red line. All participants were asked to track this line for 7 s followed by a 3-s recovery. This protocol, which lasted for 4 min, involved four sets of six intermittent isometric contractions. In order to facilitate the timing of the exercise, they have to hear two different audible metronomes that were repeated during the 4 min of the fatiguing exercise, the first beep was to start the contraction and the second beep was to stop it.

### Statistical Analysis

All data were presented as mean ± SD and were analyzed by the software Statistica 10 (Statsoft, France). Data distribution normality was confirmed with the Shapiro-Wilk test. As well, the homogeneity of the variance was verified with the Leven test. CoParea, CoP_LX_, and CoP_LY_ values were analyzed using a three-way ANOVA with repeated measures (phase × exercise × vision). IMVC; NRD for anterior, posteromedial, and posterolateral directions; and the overall YBT score were analyzed using two-way ANOVA with repeated measures (phase × exercise).

For all analyses, when ANOVA showed a significant effect, a *post-hoc* test (Bonferroni) was performed. Effect sizes (η_*p*_^2^) were calculated to assess the practical significance of our findings as recommended by Mullineaux et al. (2001). The level of significance for all statistical analyses was set at *p* < 0.05.

## Results

### Isometric Maximal Voluntary Contraction

**Figure 1:**
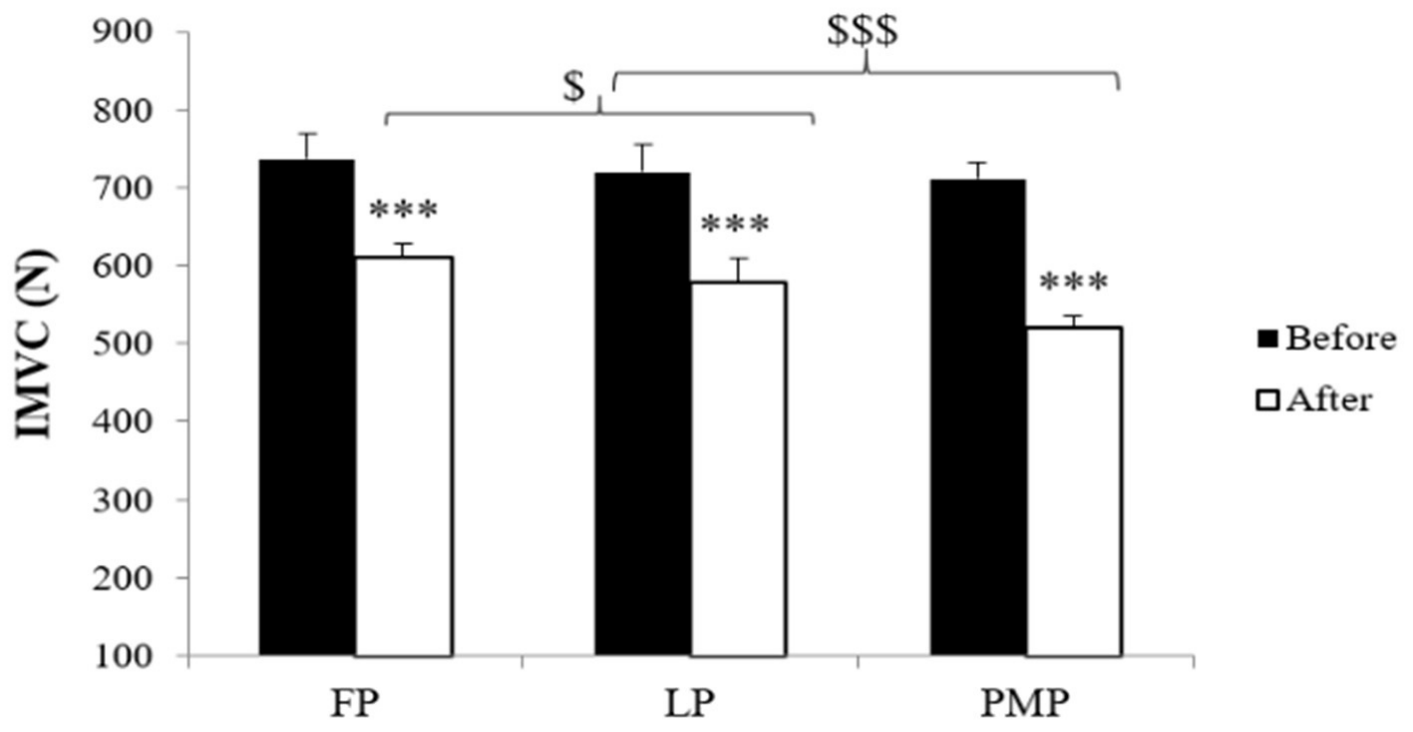
Isometric maximal voluntary contraction (IMVC) (means ± SD) during the follicular phase (FP), mid-luteal phase (LP), and premenstrual phase (PMP) before and after exercise. ^***^Significant difference compared with before exercise (*p* < 0.001), ^$^significant difference between FP and LP (*p* < 0.05), ^$$$^significant difference compared with PMP (*p* < 0.001).

The statistical analysis revealed significant main phase effect [*F*_(2, 20)_ = 28.65; *p* < 0.001; η_*p*_^2^ = 0.72], main exercise effect [*F*_(1, 10)_ = 506.25; *p* < 0.001; η_*p*_^2^ = 0.97], and main phase × exercise interaction [*F*_(2, 20)_ = 16.95; *p* < 0.001; η_*p*_^2^ = 0.60] on IMVC values. The *post-hoc* test (Bonferroni test) showed that there was no significant difference of IMVC at the preexercise among the three MCPs (*p* = 1.00 for FP compared to LP; *p* = 0.08 for FP compared PMP; *p* = 1.00 for LP compared to PMP). After exercise, IMVC values decreased significantly (*p* < 0.001) in all phases. This decrease was significantly higher in PMP compared to FP (*p* < 0.001) and LP (*p* < 0.001) and in FP (*p* < 0.01) compared to LP (Figure 1).

### Static PC

**Figure 2:**
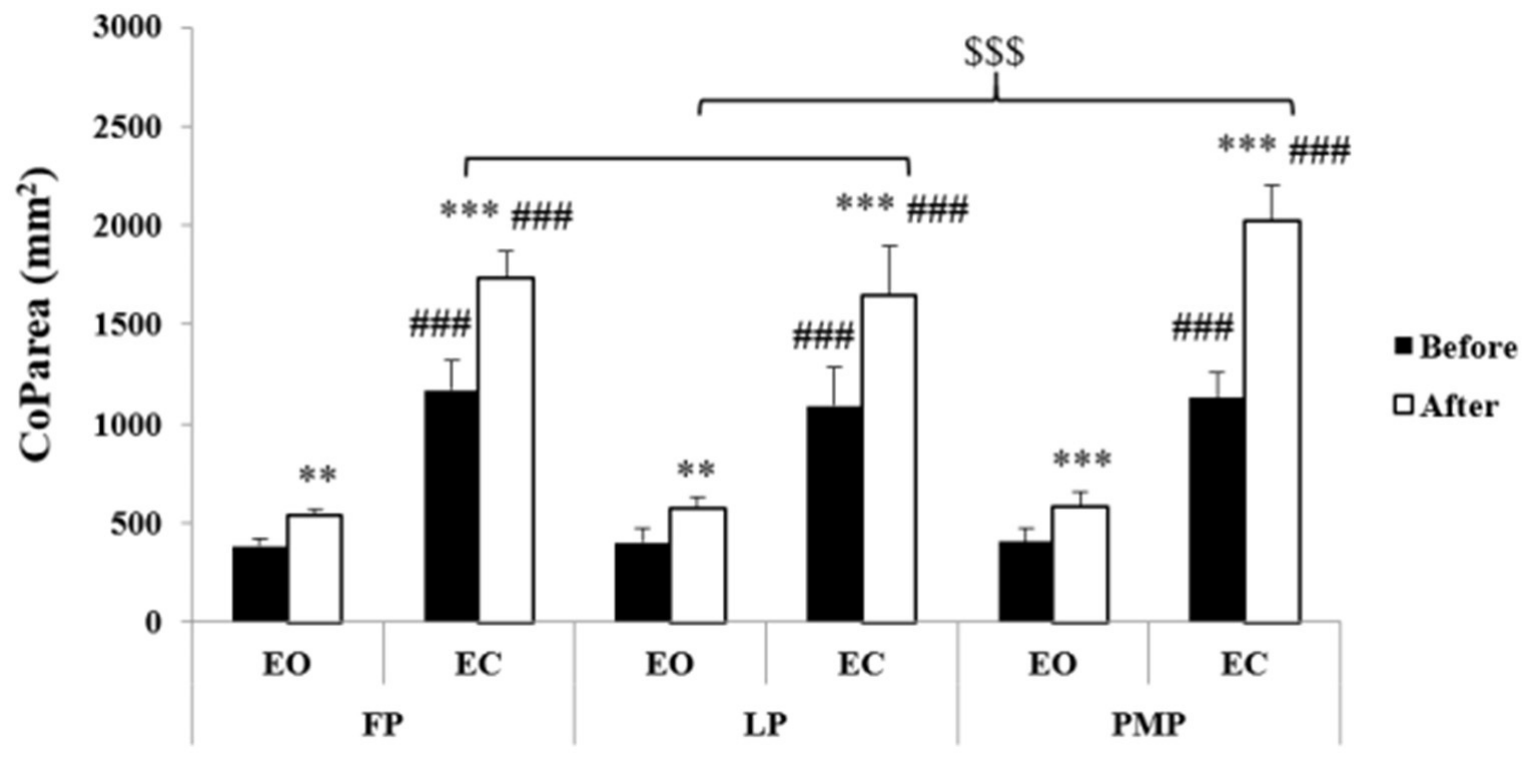
Center of pressure area (means ± SD) during the follicular phase (FP), mid-luteal phase (LP), and premenstrual phase (PMP) in the eyes open (EO) and the eyes closed (EC) conditions before and after exercise. **Significant difference compared with before exercise (*p* < 0.01), ***significant difference compared with before exercise (*p* < 0.001), ^###^significant difference compared with EO (*p* < 0.001), ^$$$^significant difference compared with PMP (*p* < 0.001).

**Figure 3:**
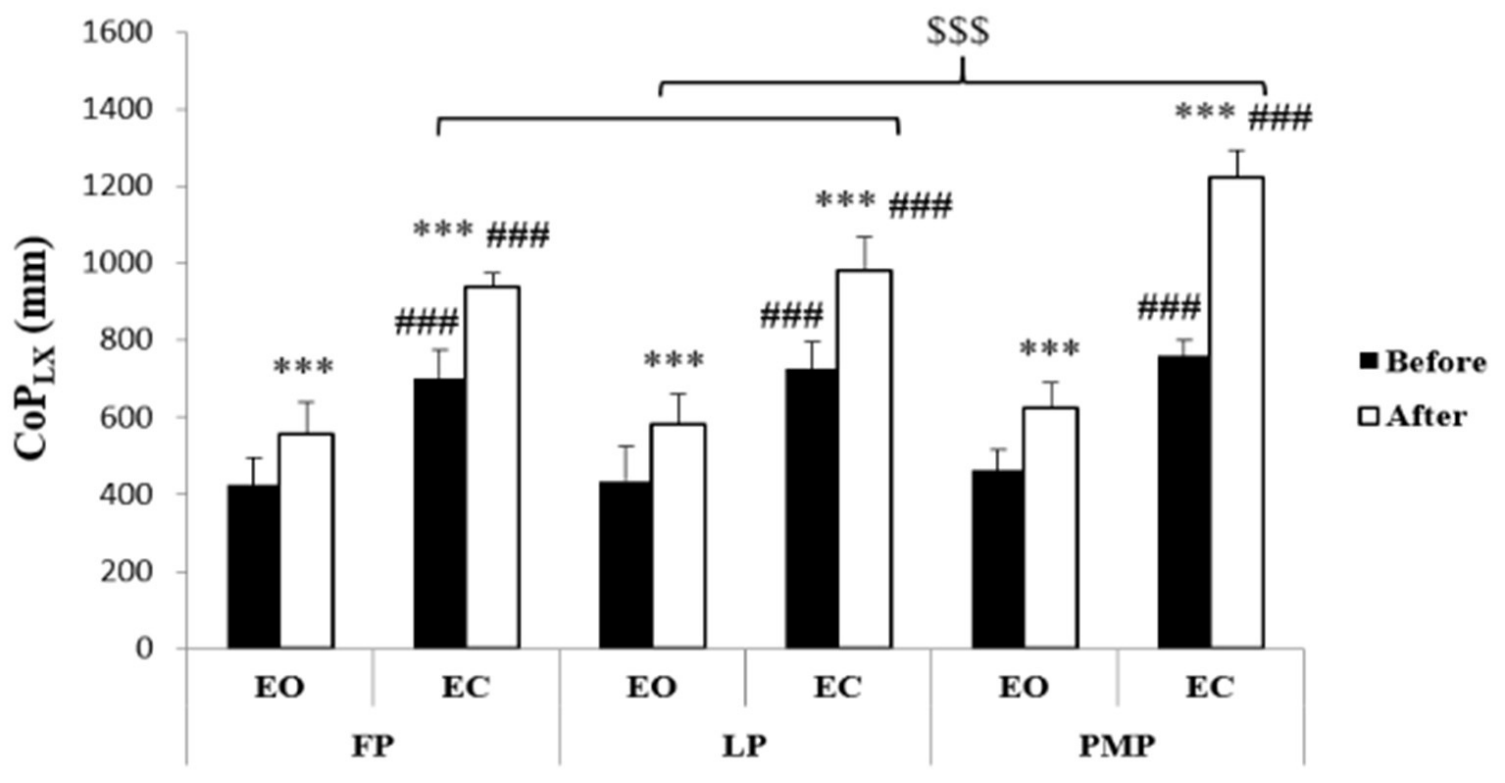
Center of pressure medio-lateral length (means ± SD) during the follicular phase (FP), mid-luteal phase (LP), and premenstrual phase (PMP) in the eyes open (EO) and the eyes closed (EC) conditions before and after exercise. ***Significant difference compared with before exercise (*p* < 0.001), ^###^significant difference compared with EO (*p* < 0.001), ^$$$^significant difference compared with PMP (*p* < 0.001).

**Figure 4:**
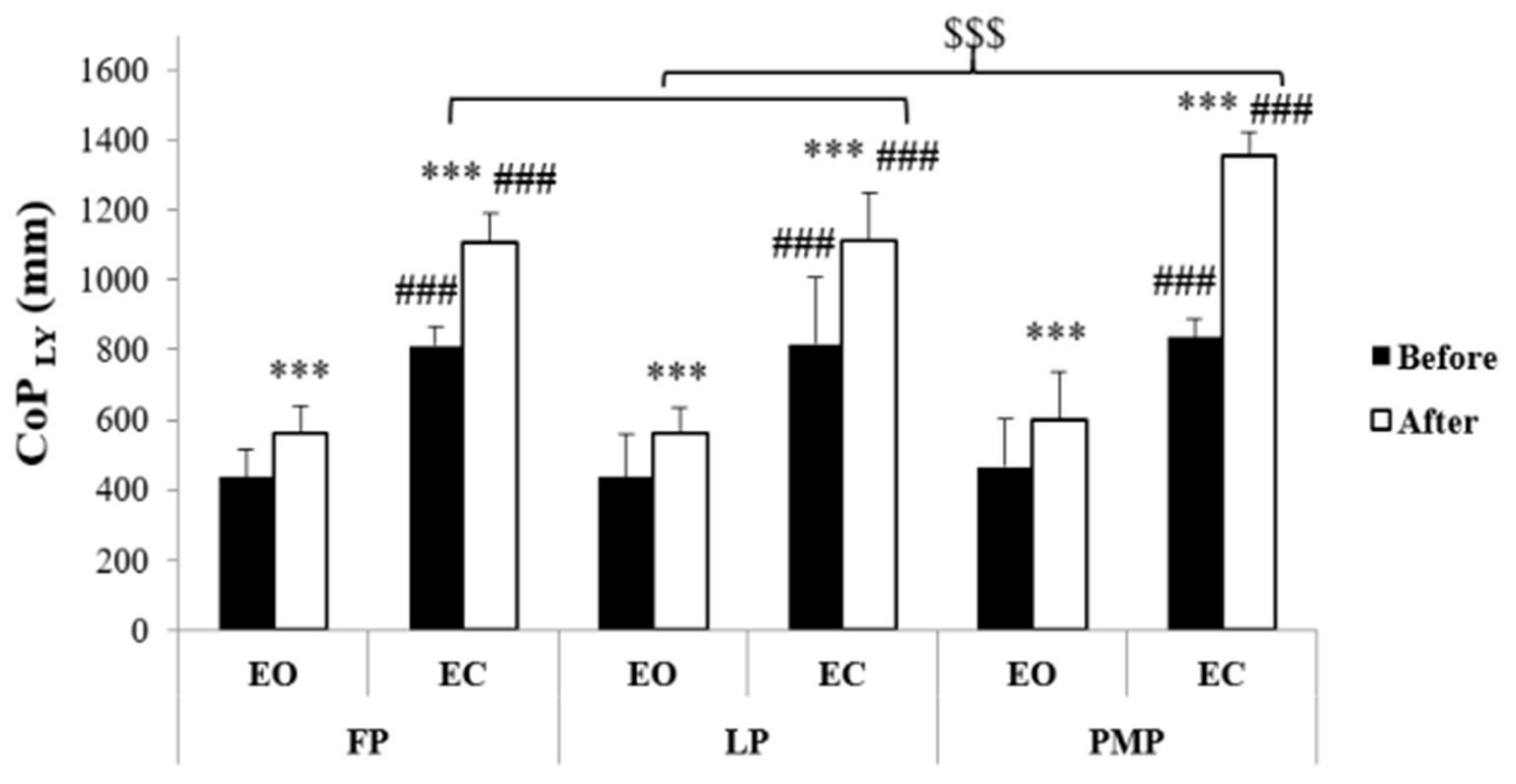
Center of pressure antero-posterior length (means ± SD) during the follicular phase (FP), mid-luteal phase (LP), and premenstrual phase (PMP) in the eyes open (EO) and the eyes closed (EC) conditions before and after exercise. ***Significant difference compared with before exercise (*p* < 0.001), ^###^significant difference compared with EO (*p* < 0.001), ^$$$^significant difference compared with PMP (*p* < 0.001).

Results of CoP parameters were presented in Figures 2–4. Statistical analysis showed significant phase, exercise, and vision main effects on CoParea, CoP_LX_, and CoP_LY_ values. Moreover, phase × vision, phase × exercise, vision × exercise, and phase × vision × exercise interactions were revealed on these parameters (Table 1). The *post-hoc* test showed that before exercise, there is no significant difference in CoParea, CoP_LX_, and CoP_LY_ values between MCPs neither in the EO (*p* = 1.00) nor in the EC (*p* = 1.00 for CoParea and CoP_LY_ between all MCP; for CoP_LX_ *p* = 1.00 between FP and LP; *p* = 0.15 between FP and PMP; *p* = 1.00 between LP and PMP) condition. Following exercise, CoParea, CoP_LX_, and CoP_LY_ increased significantly (*p* < 0.01) in all MCPs in both vision conditions. In addition, after exercise, our results showed that these values were significantly higher in the PMP compared to FP (*p* < 0.001) and LP (*p* < 0.001) in the EC condition but not in the EO one (*p* = 1.00 for CoParea and CoP_LY_ between all MCPs; for CoP_LX_ *p* = 1.00 between FP and LP; *p* = 0.45 between FP and PMP; *p* = 1.00 between LP and PMP).

**Table 1:** ANOVA results of center of pressure area (CoParea), CoP medio-lateral length (CoP_LX_), CoP antero-posterior length (CoP_LY_) values.

	**CoParea (mm** ^**2**^ **)**			**CoP**_**LX**_ **(mm)**			**CoP**_**LY**_ **(mm)**		
	***F***	***p***	***η_p_^2^***	***F***	***p***	***η_p_^2^***	***F***	***p***	***η_p_^2^***
Phase (P)	8.51	<0.01	0.43	35.5	<0.001	0.76	12.71	<0.001	0.53
Exercise (E)	1276.63	<0.001	0.99	978.78	<0.001	0.98	2016.30	<0.001	0.99
Vision (V)	1218.73	<0.001	0.99	788.97	<0.001	0.98	752.15	<0.001	0.98
P × E	19.30	<0.001	0.63	31.90	<0.001	0.74	35.33	<0.001	0.76
P × V	6.13	<0.01	0.35	20.64	<0.001	0.65	5.13	<0.05	0.31
E × V	632.26	<0.001	0.98	122.67	<0.001	0.91	198.93	<0.001	0.94
P × E × V	15.62	<0.001	0.58	23.17	<0.001	0.67	17.18	<0.001	0.60

Concerning the visual effect, participants demonstrated significantly (*p* < 0.001) higher CoParea, CoP_LX_, and CoP_LY_ in EC compared to EO condition regardless of exercise or MCP factors.

### YBT Performance

Statistical analysis showed significant main phase, exercise, and phase × exercise interaction effects on NRD for anterior, posteromedial, and posterolateral directions and the overall YBT score (Table 2). Before exercise, these NRD values for all directions as well as the overall YBT score were significantly lower in the PMP compared to FP (*p* < 0.001) and LP (*p* < 0.001) with a non-significant difference between FP and LP (NRD values for all directions, *p* = 1.00; the overall YBT reach direction score, *p* = 0.59). After exercise, all NRD values decreased significantly (*p* < 0.001) in all MCPs (Table 3). This decrease was higher in the PMP compared to FP (*p* < 0.001) and LP (*p* < 0.001) without significant difference between FP and LP (NRD for anterior and posteromedial directions, *p* = 1.00; NRD for posterolateral direction, *p* = 0.56; the overall YBT score, *p* = 0.25).

**Table 2:** ANOVA results of normalized reach distance (NRD %) for anterior, posteromedial, and posterolateral directions and the overall Y-balance test (YBT) score.

	**NRD for anterior direction**			**NRD for posteromedial direction**		
	***F***	***p***	***η_*p*_* ^2^**	***F***	***p***	***η_*p*_* ^2^**
Phase (P)	36.08	<0.001	0.76	29.37	<0.001	0.72
Exercise (E)	301.6	<0.001	0.96	284.12	<0.001	0.96
P × E	8.83	<0.01	0.44	11.03	<0.001	0.50
	**NRD for posterolateral direction**			**The overall YBT score**		
	***F***	***p***	***η_p_^2^***	***F***	***p***	***η_p_^2^***
Phase (P)	31.27	<0.001	0.73	83.85	<0.001	0.70
Exercise (E)	86.41	<0.001	0.88	451.48	<0.001	0.92
P × E	11.93	<0.001	0.52	33.22	<0.001	0.48

**Table 3:** Normalized reach distances (NRDs %) for anterior, posteromedial, and posterolateral directions and the overall Y-balance test (YBT) score in the follicular phase (FP), mid-luteal phase (LP), and premenstrual phase (PMP) before and after exercise.

	**FP**		**LP**		**PMP**	
	**Before**	**After**	**Before**	**After**	**Before**	**After**
NRD for anterior direction (%)	68.35 ± 4.35	65.91^***^± 4.54	67.86 ± 4.24	65.39^***^± 3.37	63.98^$$$^ ± 4.00	59.79^***^^$$$^ ± 3.67
NRD for posteromedial direction (%)	111.49 ± 3.99	108.62^***^± 3.73	111.33 ± 4.02	108.52^***^± 4.64	109.35^$$$^ ± 3.70	104.70^***^^$$$^ ± 4.12
NRD for posterolateral direction (%)	108.97 ± 3.84	106.11^***^± 3.45	108.48 ± 3.23	105.39^***^± 3.27	106.62^$$$^ ± 3.03	101.73^***^^$$$^ ± 3.87
The overall YBT reach direction score	96.27 ± 20.43	93.55^***^± 20.21	95.89 ± 20.48	93.10^***^± 20.25	93.32^$$$^ ± 21.36	88.74^***^^$$$^ ± 21.14

*** *Significant difference compared with before exercise (p < 0.001)*,

$$$ *significant difference compared with PMP (p < 0.001)*.

## Discussion

The current study is the first to investigate the effect of neuromuscular fatigue on PC during the different MCPs in eumenorrheic female athletes. The main results of this study showed that the disturbing effect of neuromuscular fatigue on static and dynamic PC was accentuated in the PMP compared to FP and LP. Nevertheless, the present findings demonstrated that IMVC and static PC baseline levels were not influenced by the MCP. This study's results are in line with some previous studies showing that MCs did not affect neither muscle strength (Graja et al., 2020) nor static PC (Hertel et al., 2006; Abt et al., 2007; Kaya and Çelenay, 2016) in eumenorrheic women. However, a few studies demonstrated that muscle strength (Pallavi et al., 2017; Rodrigues et al., 2019) and static PC (Fridén et al., 2003, 2005) fluctuate across the MCP. Concerning dynamic PC, this study showed that baseline YBT performance was altered in the PMP compared to FP and LP. This alteration could be explained by the impact of estrogen and progesterone concentration variations during MCP on the CNS function *via* binding to related neurotransmitters and altering their interactions (Ishii et al., 2009). In fact, female sex hormones may influence the role of the CNS and consequently the dynamic PC (Demir and Rasmi, 2015). This hormonal change may compromise the homeostasis of labyrinthine fluids, which might alter the balance ability (Demir and Rasmi, 2015). Furthermore, the decrease in estrogen level in the PMP may induce a decrease in serotonin levels and cause impaired mood, which negatively affects PC (Kaya and Çelenay, 2016). In fact, our findings are in accordance with Kaya and Çelenay (2016) who reported a higher dynamic PC alteration in menses (low estrogen and progesterone levels) compared to FP and LP without any significant difference between FP and LP. In addition, Emami et al. (2019) demonstrated that the NRD of posteromedial direction in YBT was significantly better in the ovulation phase (days 12–14; high estrogen level) in comparison to the early FP (days 3–5).

After exercise, our results showed that IMVC, CoParea, and all NRD values were altered in all MCPs suggesting that neuromuscular fatigue impaired both static and dynamic PC. Muscle force loss seems to be one of the contributing factors of static and dynamic postural impairment resulting from neuromuscular fatigue (Paillard, 2012). In fact, it was evidenced that neuromuscular fatigue deteriorates both sensory input and motor output of the postural system (Paillard, 2012). Moreover, neuromuscular fatigue is known to induce proprioceptive, vestibular, and visual sensory information disturbance (Paillard, 2012). This result is in agreement with previous studies showing that unilateral fatiguing exercises degraded static (Paillard et al., 2014) and dynamic (Whyte et al., 2015; Johnston et al., 2018) PC abilities.

The major results of our study demonstrated that the disturbing effect of neuromuscular fatigue on both static and dynamic PC was accentuated in the PMP compared to LP and FP. The accentuated amount of neuromuscular fatigue in the PMP manifested by the greater force loss after exercise could in part explain these results. Indeed, Graja et al. (2020) found a greater decrease in quadriceps muscle strength in the PMP compared to the FP and LP after high-intensity intermittent exercise and reported a significant negative correlation between the muscle force loss and the estrogen levels in eumenorrheic female athletes. As well, the correlation between force loss and PC perturbation was already demonstrated in a previous study (Borji et al., 2017). However, whether this impaired PC is due to neural or muscular figure mechanisms is still less documented. In this context, accentuated peripheral fatigue and decreased conduction velocity after fatiguing isometric contractions have been shown in PMP than other MCPs (Soares et al., 2011). According to Paillard (2012), local muscle fatigue induces muscle properties modifications including the action potential, intracellular, and extracellular ions, and other intracellular metabolites. These modifications decrease muscle excitability leading to strength loss (Paillard, 2012). The conduction velocity of afferent inputs decelerates and induces a propagation velocity decrease of the motor output, which is needed for maintaining postural balance (Paillard, 2012). Earlier researches revealed significant changes in quadriceps contractile properties and fatigability throughout the menstrual cycle (Sarwar et al., 1996). Moreover, the changes in estrogen and progesterone levels during the menstrual cycle may have an effect on neurological function (Woolley, 1999). To the best of our knowledge, the current study is the first one exploring the interaction effect of neuromuscular fatigue on PC across the different MCPs. Thus, we speculated that this accentuated alteration of CoP sway at the PMP at postexercise could be related to low estrogen level. More precisely, the low estrogen level in the PMP is associated with negative mood, which may affect the central interactions of visual, vestibular, and somatosensory inputs needed for PC (Ekenros et al., 2011). Accordingly, one could argue that the low progesterone level in cerebellar pathways occurring in the PMP may increase postural sway (Darlington et al., 2001).

Concerning the dynamic PC, the lower YBT baseline performance reported in the PMP could explain the accentuated exercise-induced impairment in this phase compared to others. The YBT has been generally used to prospectively assess injury risk (Plisky et al., 2006; Wassinger et al., 2014; Smith et al., 2015). Female athletes with an overall score of <94% are considered to be at high risk to lower extremity injury (Plisky et al., 2006). The results of the current study demonstrated that the overall YBT baseline score was lower than 94% only in the PMP and that this score decreased after fatigue in all MCPs with a greater extend in the PMP. These results suggest that the decrease of both estrogen and progesterone levels may place female athletes at a high injury risk at fresh muscle condition as well as fatigued muscle. Importantly, it has been documented that female athletes taking hormonal contraceptives had a lower injury rate, because of the high estrogen and progesterone levels in oral contraceptive pills inhibiting their variations (Nielsen and Hammar, 1991). In accordance with our results, Wojtys et al. (2002) demonstrated that the risk of injury increases a week before the start of the menstrual period in female handball players. Nevertheless, our study is the first to explore the combination between MCPs and neuromuscular fatigue effects on dynamic PC. Therefore, further studies are needed to better understand this issue. An important result could be immerged from the current study revealing that the interaction between neuromuscular fatigue and MCP effect on static PC depends on the postural task difficulty, especially the visual input availability. In fact, the disturbing effect of neuromuscular fatigue was greater in the PMP compared to the other MCP only in the EC condition. It is well-known that altered visual input increases the challenge to balance and is associated with PC perturbation (Tse et al., 2013). This is demonstrated by the higher CoP sway in the EC condition compared to the EO. Furthermore, the higher increase of CoP sway in the EC condition compared to the EO one suggests that the disturbing effect of neuromuscular fatigue on static PC was accentuated with visual input removal. Our results are in line with those of Bisson et al. (2011), who reported that the postural balance impairment related to neuromuscular fatigue was higher when vision was removed. This result could be explained by a change in sensory inflow or integration as well as a possibly altered central processing of proprioceptive input. In fact, muscle fatigue is known to hamper sensory information processing (Vuillerme et al., 2001, 2005). Besides, the visual input appears to compensate for the inordinate reafference from fatigued muscles during maintenance of upright stance (Boyas et al., 2011). Thus, vision removal increases the disturbing effect induced by neuromuscular fatigue on PC. In this context, it has been demonstrated that neuromuscular fatigue increases the Romberg's index (RI) (Borji et al., 2017), which evaluates vision contribution in PC (Njiokiktjien and Van Parys, 1976).

Our study presents some limitations. First, despite the fact that neuromuscular fatigue can be assessed by the decrease of muscle force as conducted in this current study, it will be more efficient to determine the central and peripheral contributions in the accentuated disturbing effect of neuromuscular fatigue on PC across MCPs using the twitch interpolation technique. Second, some techniques like serum tracking and profiling might be a better option to check estrogen and progesterone concentrations; but it could not be possible to use this technique due to ethical reasons. Further, in the current study, we implemented a local fatiguing exercise consisting of intermittent isometric contractions of the quadriceps muscle at 80% IMVC to explore the effect of MCP and neuromuscular fatigue on PC in female athletes. Even though local quadriceps muscle exercises were widely used in this issue (Paillard, 2012), it seems interesting to investigate other types of fatiguing exercise such as a general whole-body fatiguing protocol in further studies. Finally, our measurements were limited to the fatigued limb. Future investigations are warranted to determine if hormonal fluctuations had similar effects on both the fatigued limb and non-fatigued one or during the bipedal stance.

## Conclusion

Results of the current study suggest that baseline muscle force and static PC were not affected by the MCP hormonal variations, whereas dynamic PC was worse in the PMP compared to the other MCP in female athletes. Moreover, the disturbing effect of neuromuscular fatigue was accentuated in the PMP than the other phases, which may represent a risk factor for musculoskeletal injury in female athletes. It is worth noting to consider PC fluctuations across different MCPs when prescribing exercise programs for female athletes.

## Data Availability Statement

The raw data supporting the conclusions of this article will be made available by the authors, without undue reservation.

## Ethics Statement

The studies involving human participants were reviewed and approved by the Southern Committee for the Protection of Persons (CPP SUD) at the University Hospital (CHU) of Habib Bourgiba Sfax Tunisia. The patients/participants provided their written informed consent to participate in this study.

## Author Contributions

MK and RB conceived, designed the research, analyzed the data, edited the draft, and finalized the manuscript. MK performed the experiments. RB and SS helped to write the draft of the manuscript. HR was involved in collecting additional and further editing of the final manuscript. All authors read and approved the final version of this manuscript.

## Conflict of Interest

The authors declare that the research was conducted in the absence of any commercial or financial relationships that could be construed as a potential conflict of interest.

## Publisher's Note

All claims expressed in this article are solely those of the authors and do not necessarily represent those of their affiliated organizations, or those of the publisher, the editors and the reviewers. Any product that may be evaluated in this article, or claim that may be made by its manufacturer, is not guaranteed or endorsed by the publisher.
